# High-Accuracy Indoor Positioning and Smart Home Technologies for Assessing and Monitoring Frailty in Older Adults

**DOI:** 10.3390/s26010113

**Published:** 2025-12-24

**Authors:** Antonio Miguel Cruz, Mathieu Figeys, Yusuf Ahmed, Farnaz Koubasi, Munirah Alsubaie, Salamah Alshammari, Arsh Narkhede, Geoffrey Gregson, Andrew Chan, Lili Liu, Adriana Ríos Rincón

**Affiliations:** 1 Department of Occupational Therapy, Faculty of Rehabilitation Medicine, University of Alberta, 8205-114 St, 2-64 Corbett Hall, Edmonton, AB T6G 2G4, Canadasalamah@ualberta.ca (S.A.);; 2 Research and Innovation, Glenrose Rehabilitation Hospital, 10230 111 Avenue NW, Edmonton, AB T5G 0B7, Canadaggregson@ualberta.ca (G.G.);; 3 School of Public Health Sciences, Faculty of Health, University of Waterloo, 200 University Avenue West, Waterloo, ON N2L 3G1, Canada; 4 School of Nursing & Midwifery, College of Health, Medicine & Wellbeing, University of Newcastle, Callaghan, NSW 2308, Australia; 5 Occupational Therapy Program, College of Applied Medical Sciences, King Saud bin Abdulaziz University for Health Sciences, Alahsa 14611, Saudi Arabia; 6 Faculty of Rehabilitation Medicine, University of Alberta, Edmonton, AB T6G 2G4, Canada

**Keywords:** frailty, aging, aging in place, validation study, independent living, wireless technology

## Abstract

**Highlights:**

**What are the main findings?**

- The high-accuracy home-monitoring system demonstrated very strong concurrent validity with the Fried’s Frailty Phenotype criteria and strong associations with the Clinical and Edmonton Frailty Scales.
- The system effectively integrated ultra-wideband indoor positioning, off-the-Shelf-Internet of Things enabled devices, and smart sensors to capture all five frailty components with high spatial and temporal precision in a home-like clinical environment.

**What are the implication of the main findings?**

- High-accuracy sensor integration enables objective, continuous, and automated frailty assessment, reducing reliance on self-reported or clinic-based evaluations.
- These results show a potential for off-the-Shelf-Internet of Things-based smart home technologies to support data-driven frailty assessment and monitoring, early risk detection, and personalized intervention for aging-in-place applications.

**Abstract:**

Frailty assessment and monitoring are essential for supporting independent living and preventing adverse outcomes among older adults. This study aimed to develop and evaluate the concurrent validity of a high-accuracy home-monitoring system for assessing and tracking frailty in older adults. The system integrated off-the-shelf, zero-effort technologies, including ultra-wideband (UWB) indoor positioning, a smart scale, a connected hand dynamometer, and a Bluetooth speakerphone, to measure the five components of Fried’s Frailty Phenotype criteria. Twenty-one participants (aged 21–90 years) completed frailty assessments using both traditional clinical measures and the sensor-based system within a simulated home environment within a major rehabilitation hospital. The developed system demonstrated very strong and statistically significant correlations between the sensor-based system and the Fried’s Frailty Phenotype criteria, strong correlations with the Clinical Frailty Scale, and moderate-to-strong correlations with the Edmonton Frailty Scale, confirming the system’s strong concurrent validity. These findings indicate that high-accuracy, home-based monitoring technologies can provide reliable, objective, and non-invasive assessment of frailty in older adults, supporting early detection and continuous monitoring. This approach shows promise for future integration into smart home environments to enhance proactive frailty management and aging-in-place strategies.

## 1. Introduction

Global aging is a phenomenon driven by increasing longevity and declining fertility rates. The number of people aged 60 years and older is projected to more than double, rising from 1 billion in 2020 to 2.1 billion by 2050 [1,2]. As the number of older adults rises, the prevalence of frailty is also projected to increase. Approximately 10–15% of people aged 65 and older are considered frail, with the prevalence rising to 25–50% for those aged 85 and older [3,4]. Frailty is characterized by increased vulnerability, reduced physical reserves, and functional decline across multiple body systems [5]. As a result, frailty affects older adults’ independence, increases the risk of adverse outcomes, and raises the demand for healthcare resources [4].

The assessment of and monitoring frailty are components of frailty management among older adults; these have become increasingly important in the context of a rapidly aging global population. Information and communication technologies (ICT) offer promising, cost-effective solutions to support more proactive frailty management. By enabling continuous assessment and monitoring of frailty within the home environment, these technologies have the potential to facilitate early detection, prevention, and treatment strategies to mitigate frailty progression.

A growing body of research has explored diverse technological approaches for assessing and monitoring frailty in older adults; however, key limitations remain in the field. Wearable sensors have been amongst the most extensively investigated tools; a re-cent systematic review identified 29 studies employing 13 types of body-worn sensors, such as accelerometers, pedometers, inertial measurement units, and even smartphones affixed to the chest, to assess frailty through mobility-related indicators, including gait speed, postural transitions, step counts, and time spent walking, standing, sitting, or lying. While such devices demonstrate potential for continuous assessment and monitoring of frailty, researchers consistently report challenges related to user comfort, charging, device placement, and long-term adherence, particularly among community-dwelling older adults [6].

In response to these challenges, ambient and smart-home sensing systems have emerged as alternatives that enable passive monitoring of activities of daily living, mobility patterns, and life-space through zero- or minimal-effort technologies. These systems are embedded in the environment that automatically collect data with little to no required action or behavioral change from the user [7]. A recent review of ambient sensor systems highlighted that, while motion detectors, pressure sensors, and object-contact sensors are becoming increasingly accessible and less intrusive, most existing systems remain focused on detecting immediate risk events (e.g., falls) rather than capturing long-term frailty trajectories [8]. For instance, one study developed a so-called “Frailty Toolkit” that integrated multiple ambient sensors, including motion, door opening detectors, smart speaker, and weight scale sensors, combined with cloud-based analytics to detect room transitions, main entrance entry and exit, stair climbing, weight measurement, sitting, and exhaustion. The system demonstrated strong concurrent validity between sensor-detected activities and audio–video recordings used as the ground truth for activity recognition [9]. However, the system was not validated for frailty assessment or monitoring; it was evaluated only in a simulated laboratory environment rather than in real settings, and involved exclusively young, healthy participants. Despite these promising developments, most existing systems are limited by several key factors, including low spatial or temporal resolution, narrow sensor domains (often restricted to physical activity), intermittent data capture, and a lack of evaluation in real residential settings. Moreover, few approaches have effectively captured subtle or progressive changes across the multidimensional domains of frailty, including physical, cognitive, and social factors, in a scalable and cost-efficient manner [10,11].

A systematic review by Miguel-Cruz and colleagues identified 19 studies using in-formation and communication technologies for frailty management in older adults; however, none were suitable for real-world deployment. The mean technology readiness level reported for these systems was 6.00 (SD = 0.57; ranging from 5 to 7), corresponding to the “technology demonstration” stage. This limited level of maturity may explain why most existing research has focused primarily on the assessment and monitoring of frailty rather than on long term management and evaluation [11]. Effective frailty management would require more advanced and accurate detection systems capable of generating reliable data on which to base personalized interventions and recommendations.

In summary, despite the transformative impact of the fourth industrial revolution on healthcare through the integration of advanced information and communication technologies, the development of effective in-home frailty monitoring and management systems remains in its early stages [9,11,12]. Firstly, the majority of current frailty monitoring systems concentrate on telecommunication-based platforms implementing low-resolution uniform sensors (1.5–3 m resolution), including smartphones, tele monitoring (home monitoring), or commercially available wearable sensors and devices [13]. As a result, these systems can only measure one or two focused dimensions (proxies) of frailty, such as physical activity, postural balance, gait speed, or grip strength [9]. Since frailty is a multidimensional concept, these approaches provide limited clinical usefulness. Secondly, the adoption of these tools remains limited, as the technology readiness level of information and communication technologies for managing frailty in older adults is at technology readiness level of 6 (the technology demonstration stage), meaning they are not yet ready for real-world deployment. Finally, the evidence supporting information and communication technologies for managing frailty in older adults remains insufficient [11].

This study aimed to address this knowledge gap by developing and evaluating the concurrent validity of a high-accuracy home-monitoring system for assessing and tracking frailty in older adults (hereafter referred to as the high-accuracy home-monitoring system). It was tested against standard frailty assessments (as ground truth) administered simultaneously to determine the level of agreement between the two methods in assessing frailty. The main advance of our system, compared with earlier TRL 5–7 systems, lies in qualitative improvements, specifically, activity-based in-home assessment and the integration of all Fried’s Frailty Phenotype criteria using UWB and smart devices, rather than in achieving a higher TRL. By leveraging advanced information and communication technologies, the proposed high-accuracy home-monitoring system enables comprehensive and continuous frailty assessment and monitoring, with the potential to enhance older adults’ quality of life and reduce the burden on healthcare systems.

## 2. Materials and Methods

### 2.1. Study Design, Participants, and Setting

This study employed a correlational criterion (concurrent) validity design to evaluate a high-accuracy, in-home frailty assessment and monitoring system designed for use by older adults with or without mild cognitive impairment. Participants were recruited from the Glenrose Rehabilitation Hospital (Alberta Health Services) in Edmonton, Canada, and included older adult inpatients and outpatients, as well as adult and older adult visitors (e.g., caregivers, family members) and members of the hospital or academic community. The inclusion of adults across a wide age range allowed for representation of both robust adults and older adults with varying levels of frailty, reflecting the intended end-user population of the system, as frailty can impact all age groups (although is more prevalent in older adults).

Eligible participants met the following inclusion criteria:

(a) For inpatients or outpatients, participants were 65 years of age or older, receiving Specialized Geriatric Rehabilitation at the Glenrose Rehabilitation Hospital; for visitors or members of the hospital or academic community, participants were 18 years of age or older.
(b) All participants were able to walk independently for at least 15 m, with or without the use of a walking aid.
(c) Participants had sufficient cognitive, upper extremity, visual, and auditory function (with or without assistive devices) to interact with the smart furniture and sensors, including those living with and without cognitive impairment.

Individuals were excluded if they:

(a) had unstable cardiac conditions;
(b) were taking dopaminergic agents, cholinesterase inhibitors, anticholinergics, antipsychotics, antiepileptics, benzodiazepines and opioids that could confound frailty symptoms;
(c) had movement disorders likely to interfere with sensor data (e.g., tremors or severe spasms);
(d) had a recent viral illness (e.g., COVID-19 or influenza);
(e) required supplemental oxygen;
(f) were unable to tolerate at least one hour of moderate activity; or
(g) had insufficient English comprehension or communication ability to follow task instructions.

The participants were classified as robust, pre-frail, or frail according to Fried’s Frailty Phenotype criteria [14], supplemented by the Edmonton Frail Scale [15] and the Clinical Frailty Scale [16].

### 2.2. Setting

The study was conducted at The Independent Living Suite at the Glenrose Rehabilitation Hospital [17]. The Independent Living Suite is a fully equipped, home-like environment designed to replicate a typical residential setting, including a kitchen, bathroom, bedroom, and living area. It provides a controlled yet realistic space where patients can perform activities of daily living under professional supervision as part of their rehabilitation. This setting enables clinicians to assess functional abilities and readiness for discharge while supporting interventions that enhance safety and independence at home. Beyond its clinical role, The Independent Living Suite also functions as a living laboratory for research and innovation, offering an ideal testbed for evaluating sensor-based technologies, assistive devices, and smart home systems aimed at supporting independent living and aging in place.

### 2.3. Description of the System

**Figure 1:**
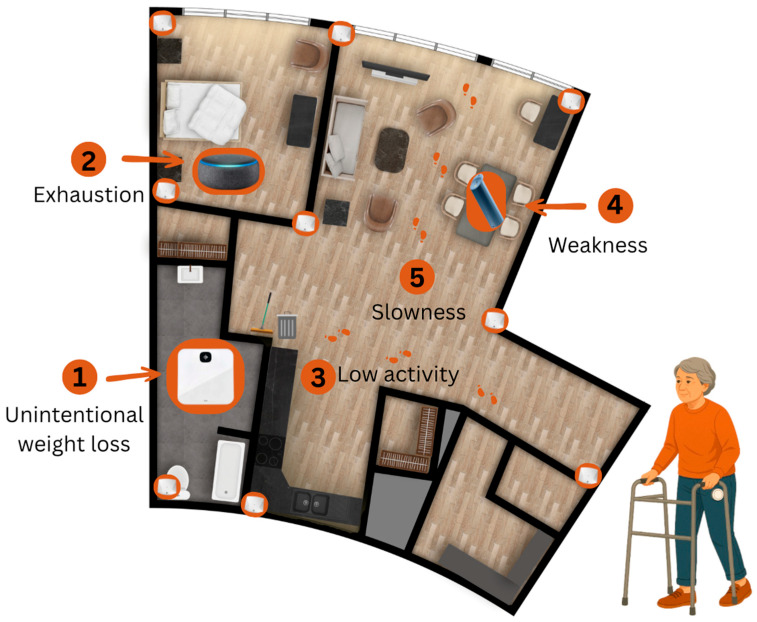
Floor plan of the Independent Living Suite, illustrating the placement of the in-home monitoring technologies.

The Product Evaluation and Application Research (PEAR) [18] Lab and the Tech4Play & Wellbeing Research Lab in the Department of Occupational Therapy, Faculty of Rehabilitation Medicine, University of Alberta, in collaboration with the Research & Innovation team at the Glenrose Rehabilitation Hospital [19] co-developed a high-accuracy in-home monitoring system for assessing and tracking frailty in older adults. A co-design phase preceded the system’s development to identify the technical specifications, desired features, and potential challenges associated with assessing and monitoring frailty in this population. During this phase, five potential users (one older adult and 4 clinicians) participated in individual interviews to inform the design process [20]. Off-the-shelf technologies were then selected and integrated to create a home-monitoring system capable of detecting all components of Fried’s Frailty Phenotype criteria. At this stage, the system has reached Technology Readiness Level (TRL) 5. TRL is a standardized scale used to describe how mature a technology is, ranging from early-stage concepts (low TRL = 1) to fully tested, proven, and ready-for-commercialization solutions (high TRL = 9). TRL 5 is defined as the integration of core technological components with realistic supporting elements and testing them in a simulated or partially realistic environment. At this stage, from one-to-several new technologies may be combined, and the system is evaluated under conditions that approximate real-world use [21,22]. In our case, we rated our system as TRL 5 because we integrated several new off-the-shelf technologies, specifically UWB indoor positioning, and three smart devices. Specifically, the UWB, a high-accuracy localization technology, had not previously been tested for frailty assessment. We evaluated the integrated system in a simulated yet realistic setting (the Independent Living Suite) with real end users. Figure 1 presents the floor plan of the Independent Living Suite, illustrating the placement of the in-home monitoring technologies. The system consisted of:

- A Pozyx^®^ indoor positioning system (Pozyx, Belgium, Ghent [23]) equipped with ultra-wideband (UWB) technology to monitor low physical activity and slowness (walking time) variables of Fried’s Frailty Phenotype criteria. It involves the installation of nine UWB anchors placed in the corners of each room of the Independent Living Suite. The user dons a wearable tag that communicates with the anchors and the system transmits (when more than 3 anchors are used) [24] its position to provide furniture-level resolution on activities in the Independent Living Suite. This high-resolution tracking (up to 10 cm indoor positioning accuracy) allowed for meaningful continuous indoor monitoring, as well as identifying the areas of the Independent Living Suite that the participant occupies with corresponding durations. A detailed description of the sensor data format and specifications has been fully published elsewhere [25].
- A K-Grip^®^ device, an Internet of Things connected dynamometer to measure weakness (grip strength) of Fried’s Frailty Phenotype criteria [26].
- A Bluetooth Speakerphone AIRHUG 01^®^ (100 Hz–24 kHz) to collect the self-report exhaustion of Fried’s Frailty Phenotype criteria [27]. This device is equipped with an upgraded full-duplex digital microphone, which can pick up voice within a 6 m radius.
- A Fitbit Aria Air™, a smart weight scale for tracking shrinking (Unintentional weight lost) of the Fried’s Frailty Phenotype criteria [28]. This is an easy-to-use smart scale that displays the participant’s weight and syncs it to the Fitbit app.

Data was accessed through a password-protected web portal that uses ngrok Inc. (San Francisco, CA, USA) to create a secure tunnel to our server and Grafana Labs© (New York, NY, USA) to visualize and export data [29,30]. The ngrok service creates secure tunnels to our locally hosted application using a reverse proxy. It uses Transport Layer Security 1.3 protocol to authenticate and encrypt connections between web browsers and our web server.

### 2.4. Variables, Standard Measures, and Sensors

The primary outcome of this study was frailty status, using the phenotypic approach, assessed using Fried’s Frailty Phenotype criteria: unintentional weight loss, exhaustion, physical activity, weakness, and slowness [14]. Fried’s Frailty Phenotype uses a dichotomous scoring system for each criterion (1 = meets frailty criterion; 0 = does not meet criterion), according to established thresholds, and an overall frailty score ranging from 0 to 5 based on the sum of these criteria. We applied the same scoring structure to our sensor-based assessments, generating both per-criterion dichotomous scores and a total sensor-based frailty score (0–5). We chose Fried’s five-component frailty phenotype scale as it is commonly applied clinically, is quick and objective to administer, and is easily adaptable for sensor-based measurements. It is also the most widely used framework in information and communication technology-based studies of frailty, allowing for comparability with previous research [11].

In addition to Fried’s Frailty Phenotype criteria, the Edmonton Frailty Scale (EFS) and the Clinical Frailty Scale (CFS) were administered to provide complementary assessments of frailty. The EFS is a validated and reliable multidimensional tool developed at the University of Alberta that evaluates nine domains relevant to frailty: cognition, general health, functional independence, social support, medication use, nutrition, mood, continence, and functional performance. Total scores range from 0 to 17, with higher scores indicating greater frailty [15]. The CFS is a clinician-rated, 9-point global measure of frailty that reflects overall health status, functional abilities, mobility, and level of independence, ranging from 1 (very fit) to 9 (terminally ill) [16]. Together, these scales capture broader clinical, cognitive, psychosocial, and functional aspects that are not encompassed by Fried’s Frailty Phenotype criteria alone. We incorporated the overall EFS and CFS scores to support the criterion validation of the high-accuracy home-monitoring system against established clinical measures.

#### 2.4.1. Unintentional Weight Loss

Unintentional weight loss refers to an involuntary reduction in body weight [31]. It reflects underlying physiological decline, including loss of muscle mass and energy reserves, and is strongly associated with reduced strength, impaired immunity, and increased risk of disability, hospitalization, and mortality, making it a key indicator of frailty progression [32].

Non-sensor measurement (ground truth). Participants were weighed using a standard analog or digital scale. They were also asked if they had unintentionally lost more than 4.5 kg (10 lbs) or 5% of their body weight in the past year [14].

Sensor-based measurement: A smart scale (Fitbit Aria Air™) recorded participant body weight, syncing the data to a secure platform. The voice-controlled smart speaker Bluetooth Speakerphone AIRHUG 01^®^ was configured with Google Speech to Text API to ask some predefined questions without requiring a wake word. The speaker asked participants whether they experienced unintentional weight loss using structured questions with predefined response options (“yes” or “no”). Responses were captured, processed, and stored securely on a local server connected to a password-protected web portal. These verbal responses, together with the weight data, were used to determine frailty status.

Fried’s frailty criterion: For both non-sensor and sensor-based assessments, the unintentional weight loss criterion, based on Fried’s Frailty Phenotype, was defined as reporting an unintentional loss of more than 5% of body weight or more than 4.5 kg over the past year [14]. Binary coding was applied where 1 = present; 0 = absent.

#### 2.4.2. Exhaustion

Exhaustion refers to persistent fatigue or a lack of energy and is a key component of frailty, as it indicates a reduced physiological reserve and a diminished ability to cope with everyday physical demands, thereby increasing vulnerability to functional decline and adverse health outcomes [5].

Non-sensor measurement (ground truth): In this study, two items were used from the Center for Epidemiological Studies–Depression Scale (CES–D) to measure exhaustion, i.e., “I felt that everything I did was an effort” (item 7 of the CES-D scale) and “I could not get going” (item 8 of the CES-D scale). The CES–D is a 10-item self-report tool designed to measure depressive symptoms in the general population [33]. The CES-D assesses the frequency of symptoms experienced during the past week, with responses scored from 0 (rarely) to 3 (most of the time). The total score ranges from 0 to 30, with a score of 10 or greater used as the cut-off for depression. The CES–D has demonstrated good reliability (Cronbach’s α > 0.85) and construct validity across diverse populations, including older adults [34]. It has been frequently used in geriatric research to assess depressive symptoms and as part of frailty definitions, particularly for measuring exhaustion [35]. Prior studies have shown that these two items specifically correlate with fatigue and reduced physical function in older adults [35,36]. Depression and exhaustion are closely linked: depressive symptoms, such as low energy and lack of motivation, often manifest as feelings of effortlessness and difficulty initiating activities [37]. In older adults, this overlap contributes to functional decline and increased vulnerability, reinforcing the role of depressive symptoms as a marker of frailty [38].

Sensor-based measurement: The Bluetooth Speakerphone AIRHUG 01^®^ in the bedroom asked participants the two CES–D items. Responses were captured via voice and analyzed to determine frailty classification. This task has been validated in a previous study [9]. The conversation was initiated when other sensors detected the participant’s activity in the Independent Living Suite bedroom. The interactive smart speaker asked three questions that accept single-word answers.

1. “Are you available to answer questions at the moment?” If the user answered “yes”, then, the system proceeded to question 2.
2. “Do you feel that everything you did was an effort, or you could not get going in the last week?” Answer with yes or no.
3. Third question: “How often in the last week did you feel this way?”. Answer options “1–2 days” or “2 or more days.”
4. A chatbot was programmed with a fallback plan to repeat questions if the first attempt was not successful.

Fried’s frailty criterion: For non-sensor measurement, exhaustion in Fried’s frailty phenotype was assessed based on the frequency of self-reported statements such as “I felt that everything I did was an effort” or “I could not get going.” A response of 2 (moderate amount of the time) or 3 (most of the time) to either of the selected items from the CES-D scale [33] classifies the participant as frail on this component [14]. For sensor-based measurement, responses are coded as 0 for “0 to 2 days” and 1 for “3 to 7 days.” A code of 1 indicates that the participant meets the frailty criterion (Present) for exhaustion, while a code of 0 indicates that they do not (Absent).

#### 2.4.3. Physical Activity

Physical activity is defined as the level of energy expenditure during leisure and household activities. Low physical activity is associated with frailty, as insufficient activity leads to reduced muscle mass, strength, and endurance, increasing vulnerability to functional decline, disability, and adverse health outcomes [39,40].

Non-sensor Measurement (ground truth): The Fried Frailty Phenotype utilized the Minnesota Leisure Time Physical Activity Questionnaire (MLTPAQ), however, in this study, the CHAMPS was used for several reasons; the MLTPAQ is less suited to contemporary older adult cohorts [41], whereas the CHAMPS was specifically validated for older adults; in addition, the researchers were unable to retrieve the MLTPAQ utilized in the study conducted by Fried and colleagues [14]. The Community Healthy Activities Model Program for Seniors (CHAMPS) questionnaire assessed weekly frequency and duration of various activities [41]. It is a validated self-report instrument designed to measure the weekly frequency and duration of 41 physical and social activities commonly performed by older adults. The CHAMPS has demonstrated good test–retest reliability (ICC ranging from 0.58 to 0.62) and construct validity when compared with objective measures like accelerometers and physical performance tests. It has been used in geriatric research to assess physical activity levels, monitor intervention outcomes, and identify individuals at risk for frailty due to inactivity [42].

The CHAMPS provides measures of both weekly frequency and estimated weekly caloric expenditure (kcal/week) in physical activity. For this study, we used the kcal/week measure for all exercise-related activities (28 activities in total). Caloric expenditure per week was calculated according to the procedures outlined in the CHAMPS codebook [41] as follows: First, self-reported activity durations were recoded into standardized categories representing hours per week (0, 0.5, 1.75, 3.75, 5.75, 7.75, or 9.75). Activities reported as not undertaken were coded as zero. Each recoded duration was then multiplied by the corresponding metabolic equivalent of task (MET) value to obtain a MET-hours/week score [43]. Caloric expenditure for each activity was calculated by multiplying this score by 3.5, by 60 (to convert to MET-minutes), and by the participant’s body weight in kilograms divided by 200. Total weekly caloric expenditure was then derived by summing the energy expenditure values across all reported activities. Equation 1 summarizes these steps.

(1) $${kcal}_{total}=\sum_{i=1}^{n}{[D_{i}*MET}_{i}*3.5*60*(\frac{W}{200})]$$

where

i: Activities conducted by the participant (1…n);

n: All exercise-related activities (28 activities);

Di: recoded hours/week for activity (recoded as: 0, 0.5, 1.75, 3.75, 5.75, 7.75, 9.75);

METi: Metabolic Equivalent of Task (MET) value of each activity;

W: Participant’s body weight (kg).

This total caloric expenditure was then compared against sex-specific cutoffs (<383 kcal/week for men and <270 kcal/week for women) to determine whether participants met the frailty criterion for low physical activity, as described by Fried and colleagues [14].

Sensor-based Measurement: Examples of CHAMPS exercise-related activities include dancing, playing golf or tennis, skating, household chores such as sweeping, and gardening. Many of these activities cannot be performed safely within a controlled home-like environment such as the Independent Living Suite, and several are interest-dependent, meaning individuals engage in them based on personal preference rather than routine necessity. For these reasons, we sought alternative sensor-based measures using instrumental activities of daily living that are commonly performed by adults across genders and ages, fall within light (<3 METs) or moderate (3–6 METs) intensity, and can be safely completed by individuals who may be frail [43]. The research team selected two such tasks—“Taking out the garbage” and “Sweeping”—from the Performance Assessment of Self-Care Skills (PASS) [44]. As per PASS instructions, taking out the garbage involved gathering a standardized garbage bag (approximately 2–4 kg), walking it approximately 10 feet to a designated disposal area within the Independent Living Suite. Sweeping involved using a broom to clean a defined 1.5 × 1.5 m floor area, sweeping a dry material (dry cereals) into a dustpan, and then transferring it into a garbage can [44]. The PASS is a validated, performance-based measure that evaluates an individual’s ability to carry out functional tasks by observing independence, safety, and adequacy [44]. The PASS has demonstrated excellent interrater reliability (ICC > 0.90) and test–retest reliability (ICC > 0.80), as well as strong content and construct validity across diverse populations, including older adults and individuals with cognitive impairment [45]. Motion trajectories, duration, rest periods, and speed were recorded using UWB positioning technology.

Fried’s frailty criterion: For the non-sensor-based frailty measure, we applied Fried’s cutoff, which defines frailty as being in the lowest 20% of weekly caloric expenditure (<383 kcal/week for men and <270 kcal/week for women). This was coded as a binary variable, with 1 indicating frailty due to low physical activity and 0 indicating otherwise [14].

Sensor-based frailty classification was based on the total duration (s) across the two PASS tasks. We calculated the 80th percentile values to establish thresholds for identifying low physical activity. Using the 80th percentile for task completion time means that 80% of participants completed the activities more quickly, while the slowest 20% had the longest times, that is paralleling the reference framework in which the lowest-performing 20% in Kcals expenditure per week indicate frailty. Participants were coded as 1 (meets frailty criterion) or 0 (does not meet frailty criterion) accordingly.

#### 2.4.4. Weakness

Weakness, defined as reduced muscle strength, was assessed through grip strength, an objective indicator of overall muscle function and health [46,47]. Lower grip strength reflects reduced muscle mass and functional capacity, and is linked to a higher risk of frailty, as it is associated with mobility limitations, loss of independence, and a greater likelihood of disability and adverse health outcomes [48].

Non-sensor Measurement (ground truth): Grip strength was measured using a Baseline Lite hydraulic hand-held dynamometer (200 lb or 91 kg capacity). Participants were instructed by a research assistant to squeeze the device as strongly as possible for each trial, performing three trials per hand with a 30 s rest between trials. The research assistant recorded the peak grip measure for each trial.

Sensor-based Measurement: Participants used the K-Grip^®^ dynamometer to assess handgrip strength following the same instructions as per the non-sensor measurement. The device recorded the peak grip strength in real-time and transmitted the results to the data platform.

Frailty Criteria (Grip Strength Cutoffs by Sex and Body Mass Index (BMI)): For both non-sensor and sensor-based assessments, frailty due to weakness was assessed using grip strength cutoffs specific to sex and BMI according to Fried et al. 2001 [14]. Table 1 shows the sex- and BMI-specific grip strength thresholds used to classify frailty due to weakness, with lower cutoffs applied to individuals with lower BMI. For both the non-sensor and sensor-based assessments, weakness was then coded as a binary variable, with 1 indicating that the frailty criterion was met (present) and 0 indicating that it was not met (absent).

#### 2.4.5. Slowness

Slowness is characterized by reduced gait speed and is associated with decreased mobility and higher frailty [49]. As a slower walking speed reflects diminished muscle strength, balance, and endurance, it increases the risk of disability, loss of independence, and adverse health outcomes [50].

Non-sensor Measurement (ground truth): The total time (s) to walk 15 feet was used to assess slowness [14]. This test is a simple performance-based measure in which participants are asked to walk a distance of 15 feet (4.57 m) at their usual pace, and the time taken is recorded manually using a stopwatch. This test is used to evaluate gait speed, an important indicator of functional status and overall health in older adults. The 15-foot walk test has demonstrated good test–retest reliability (ICC > 0.90) and strong concurrent validity through significant correlations with other mobility and physical performance measures, such as the 5 m walk test and the Timed-Up-and-Go test. It has also shown predictive validity for adverse health outcomes, including disability, hospitalization, and mortality in older adults [51].

Sensor-based Measurement: The UWB indoor positioning system tracked walking time and movement trajectory. Walking time was measured using a UWB indoor positioning system, which tracked participants’ movement trajectories with sub-10 cm accuracy. The sensor collected x, y, and z coordinates along with Unix timestamps. The coordinates were used to calculate the Euclidean distance traveled using the distance between two points formula, while the Unix timestamps were converted to seconds and adjusted to the UTC-7 time zone. These time and distance values were then plotted in Excel, where a regression line (y = bx + c) was generated for each participant, with distance as the dependent variable (y) and time as the independent variable (x). The coefficients b (slope) and c (intercept) differed for each participant. To estimate the time taken to walk 15 feet, 15 was substituted for y (distance), and the corresponding x (time) was calculated using the regression equation.

The UWB, by design, does not perform any data processing beyond presenting the raw, positioning data after triangulation from the engaged anchors. These raw data are in x, y, and z coordinates with a time stamp. To calculate the actual distance traveled (and then the speed—Speed = Distance/Time), the Distance between two points function has to be employed. However, this function only produced an accurate result if the path is perfectly linear. Even though in the experiment, a linear path of 15ft was marked on the floor for the participants to walk, most of them followed their own mean path, though within the average of the actual line mark, creating a zigzag movement. Older adults often cannot maintain a straight walking path due to muscle weakness, balance and/or gait instability. Hence, a regression model was sought to find the line of best fit that can be the closest representation of the path taken and distance covered by the participants, reducing small location errors and errors due to the zigzag movement.

Frailty criterion (Time to Walk 15 Feet): For both non-sensor and sensor-based assessments, frailty due to slowness was assessed using the time taken to walk 15 feet, with cutoffs based on sex and height according to Fried and colleagues’ study. For men, the threshold was ≥6 s for those taller than 173 cm and ≥7 s for those 173 cm or shorter. For women, the threshold was ≥6 s for those taller than 159 cm and ≥7 s for those 159 cm or shorter. Slowness was coded as a binary variable, where 1 indicated meeting the frailty criterion and 0 indicated not meeting it [14].

#### 2.4.6. Frailty Total Score

Frailty total scores for both non-sensor-based and sensor-based assessments were calculated using Fried’s five-criteria frailty phenotype criteria [14]. According to this calculation, a total score of 0 indicates a robust status, scores of 1–2 indicate a pre-frail status, scores of 3–4 indicate frailty, and a score of 5 indicates very frail status.

#### 2.4.7. Sociodemographic, Cognitive, and Functional Measures

Participant characteristics included demographic information (e.g., age, sex, gender identity, educational attainment, and medical history), technology literacy (frequency and types of digital technology use), cognitive status, assessed using the Saint Louis University Mental Status Examination (SLUMS), and level of independence using the Barthel Index of Activities of Daily Living [52,53].

### 2.5. Ethics

Ethics approval was obtained through the University of Alberta Health Research Ethics Board—Health Panel (Pro00131722) with operational approvals obtained through the Northern Alberta Clinical Trials and Research Centre (NACTRC). The study was registered prior to commencing on clinical-trials.gov (NCT05961319). All participants signed an informed consent form. In instances where participants lacked the capacity to provide written consent, we identified and obtained consent from appropriate alternate decision-makers, such as legal guardians, family members, or authorized representatives.

### 2.6. Data Collection Procedures

Recruitment took place between 20 September 2023, and 20 March 2024. Eligible inpatients were identified by nurses at two specialized geriatric rehabilitation units (i.e., 3D and 4C) at the Glenrose Rehabilitation Hospital, while the remaining participants were recruited through word-of-mouth and snowball methods. Individuals interested in participating then contacted the research team. Individuals who met the selection criteria were invited to participate. After obtaining consent, data collection began. Data collection followed a two-stage process:

#### 2.6.1. Stage 1: Frailty and Baseline Assessment

A research assistant administered a demographic and health questionnaire, as well as the Fried’s Frailty Phenotype criteria [14], using standard instruments (e.g., CES–D, CHAMPS, Barthel Index), the CFS, and the EFS. In addition, research assistants assessed grip strength using a standard dynamometer, weight using a standard digital scale, and administered the 15-foot walk test.

#### 2.6.2. Stage 2: Sensor-Based Tasks

Participants performed seven scripted tasks while interacting with the high-accuracy home-monitoring system:

- Participants weighed themselves using the Fitbit Aria Air™ scale in the Independent Living Suite bathroom.
- In the bedroom, participants responded to questions delivered by the Bluetooth Speakerphone AIRHUG 01^®^ regarding unintentional weight loss and exhaustion.
- In the kitchen, participants completed two PASS tasks: taking out the garbage and sweeping.
- In the dining room, participants squeezed the K-Grip^®^ device while seated at the dining table.
- Participants then walked 15 feet in a straight trajectory through the clear space between the dining and living rooms (see Figure 1).

Sensor data (e.g., location, interaction time, strength metrics, movement trajectories) were collected and timestamped.

### 2.7. Statistical Methods

Descriptive statistics summarized participant demographics and clinical variables. Means and standard deviations were calculated for continuous variables; frequencies and proportions were reported for categorical variables. To evaluate the criterion validity of the sensor-based home-monitoring system, we applied both Spearman’s rho and Cohen’s kappa. Spearman’s rho was used to examine the correlations between the total frailty scores generated by the high-accuracy home-monitoring system and those obtained from Fried’s Frailty Phenotype, the Edmonton Frailty Scale (EFS), and the Clinical Frailty Scale (CFS) (score ranges: Fried’s Phenotype and home-monitoring system = 0–5; EFS = 0–17; CFS = 1–9). Spearman’s rho was also applied to assess the correlations between the dichotomous scoring of each of the five criteria in Fried’s Frailty Phenotype [15] (1 = meets frailty criterion; 0 = does not meet frailty criterion) and the corresponding dichotomous scores derived from the home-monitoring system. In addition, Cohen’s kappa was used to evaluate the level of agreement between dichotomous frailty classifications across the two methods for each of the five criteria. Although no universal standard exists for interpreting correlation coefficients (r), commonly used guidelines in medicine, biomedicine, healthcare, sociology, and psychology classify |r| < 0.3 as a weak relationship, 0.3 ≤ |r| ≤ 0.5 as a moderate relationship, and |r| > 0.5 as a strong relationship [54].

**Figure 2:**
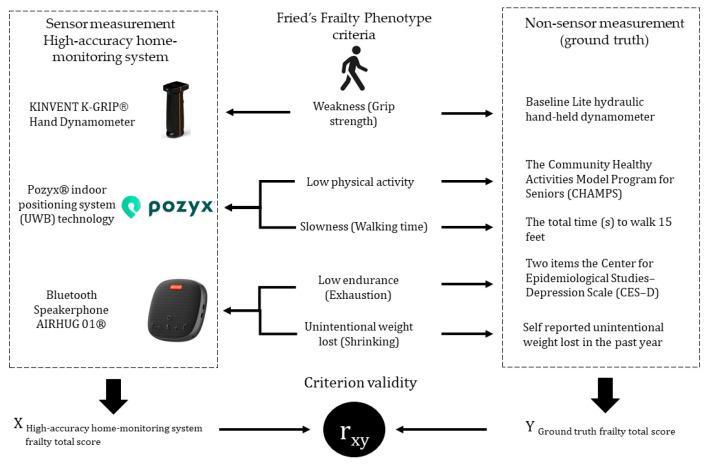
Overview of Data Collection and Analysis.

In addition, the total Frailty score from the Fried’s Frailty Phenotype and the high-accuracy home-monitoring system were recorded as a dichotomous classification, with scores of 0–2 (robust to pre-frail) categorized as non-frail and scores of 3–5 (frail to very frail) categorized as frail. We applied both Spearman’s rho and Cohen’s kappa to this dichotomized total score of frailty. A significance level of *p* < 0.05 was applied for all statistical tests. Analyses were conducted using the Statistical Package for Social Sciences 2.9 (SPSS Inc., Chicago, IL, USA). Figure 2 provides an overview of the study procedures, measures, and data analysis.

## 3. Results

### 3.1. Participants’ Description

The study sample comprised 21 participants, consisting of 11 males (52.4%). Overall, the mean age was 51.86 years (SD 26.48), with the most common age group being older than 60 years (52.4%). Most participants held a bachelor’s degree (33.3%), followed by a high school diploma (28.6%). The majority reported English as their first language (90.5%), lived in the community with others (66.7%), and were right-handed (95.2%). A total of 57.1% of participants were community members. Polypharmacy (i.e., the concurrent use of multiple medications) was reported by all inpatients, while only one non-inpatient participant—a male—reported polypharmacy. On baseline assessments, the mean SLUMS score was 27.81 (SD 3.01), indicating that participants were, on average, within the normal cognitive range [53]; however, scores for five participants suggested the presence of mild cognitive impairment. The mean Barthel Index score was 18.95 (SD 1.59), indicating that most participants were highly independent in basic activities of daily living. However, nine (43%) participants had scores suggesting slight dependence, requiring minimal assistance with certain basic daily activities [52]. There were several notable sex-related differences. Females were considerably older than males (mean age 61.10 vs. 43.45 years), and they represented the majority of inpatients (70.0%). Males demonstrated greater physical strength and stature, showing higher maximum grip strength in both the dominant (38.87 kg vs. 27.04 kg) and non-dominant (37.60 kg vs. 22.02 kg) hands, and were taller on average (176.18 cm vs. 164.76 cm) (See Table 2 for more details).

The technology literacy profile of the 21 participants (see Table S1 in Supplementary Materials) revealed high engagement with commonly used devices, such as smartphones and computers, while the use of newer or less essential technologies was more variable or infrequent. The majority reported daily use of smartphones (85.7%) and computers (81.0%). Use of smart TVs was more heterogeneous, with 47.6% using them daily and 23.8% reporting no use. For use of tablets, 33.3% of participants reported never using one, and 28.6% showed daily use. Engagement with emerging technologies was notably lower: 57.1% never used a smartwatch, and 52.4% used smart home devices only a few times per year. Among older adults, daily use was reported by 73% for smartphones, 64% for computers, 55% for smart TVs, 36% for tablets and smart home devices, and 27% for smartwatches.

The participants’ frailty profile, assessed using three different scales (Table S2 in Supplementary Materials), generally indicated a healthy group, though the results varied depending on the metric used. When using the Fried’s Frailty Phenotype criteria, administered as the ground truth for this study, over half of the participants (11 participants, 52.4%) were categorized as Pre-frail, while 33.3% were Robust and 14.3% were Frail. According to the EFS, the vast majority of the sample (17 participants, 80.9%) were classified as Not frail, with only 19.1% considered Vulnerable, and no participants falling into the categories of mild, moderate, or severe frailty. Similarly, the CFS showed that most participants were in good health categories, with 28.6% classified as Well and 26.6% as Very fit; only 4.8% were categorized as Mildly frail, with no one categorized in the more severe levels. Both the EFS and the CFS are based on self-report.

### 3.2. System Validation

Table 3 presents the comparison between frailty classifications derived from the standard Fried’s Frailty Phenotype criteria (ground truth) and those obtained using the high-accuracy home-monitoring system among the 21 participants. Overall, the high-accuracy home-monitoring system identified more cases of frailty than the ground truth across most components. Specifically, it detected more cases of low physical activity, weakness, and slowness, with the largest discrepancy observed in slowness, where the high-accuracy home-monitoring system measure identified roughly twice as many cases as the standard method. In contrast, the ground truth identified more cases of poor endurance. Consequently, the overall frailty classification based on the high-accuracy home-monitoring system yielded to two more number of participants classified as frail compared to Fried’s Frailty Phenotype criteria.

The reliability coefficients (Cohen’s Kappa) comparing the two methods varied notably across frailty components. High and statistically significant agreement was observed for unintentional weight loss (shrinking) and weakness. Slowness showed moderate but significant agreement. In contrast, low physical activity and exhaustion demonstrated low and non-significant agreement. Overall, the dichotomized frailty classification showed moderate-to-substantial and statistically significant agreement between the high-accuracy home-monitoring system and the Fried’s Frailty Phenotype criteria.

Correlations (Spearman’s rho) between the ground truth and the high-accuracy home-monitoring system measurements ranged from very strong to negligible. Consistent with the reliability findings, unintentional weight loss (shrinking) demonstrated a perfect correlation. Weakness also showed a very strong and statistically significant correlation. Slowness exhibited a moderate significant correlation, while low physical activity showed a weak-to-moderate correlation, approaching significance. In contrast, low endurance (exhaustion) demonstrated a negligible and non-significant correlation. The total frailty score, categorized as robust (score = 0), pre-frail (score = 1–2), and frail (score = 3–4), demonstrated moderate and statistically significant agreement, as well as a strong, statistically significant correlation between the high-accuracy home-monitoring system and Fried’s criteria. When the dichotomized classification was applied, agreement between the two methods increased to a substantial and statistically significant level, and the correlation remained strong and statistically significant.

Specifically, for the low physical activity criterion, an additional analysis was conducted to clarify the empirical correspondence between Fried’s cutoff, which defines frailty as being in the lowest 20% of weekly caloric expenditure (from CHAMPS in our study), and the slowest 20% of participants (those with the longest completion times) in two instrumental activities of daily living (80th percentile). A cross-analysis comparing low physical activity classifications derived from both systems was performed for the entire sample and stratified by age and sex. Overall, the results demonstrated good agreement: the high-accuracy home-monitoring system and the CHAMPS concurred in classifying 17 of 21 participants (81%), including 15 as robust and 2 as frail.

When stratified by age, performance differences emerged. The system demonstrated perfect agreement in the younger group, correctly classifying all 10 participants as robust. Among older adults, the high-accuracy home-monitoring system and the CHAMPS agreed in classifying 7 of 11 individuals (64%) (five robust and two frail) (Cohen’s kappa (κ), (Young Adults) κ = 1.00, *n* = 10, *p*, no statistics are computed because perfect agreements between methods; Cohen’s kappa (κ), (Older Adults) κ = 0.241, *n* = 11, *p* = 0.387). Sex-stratified analyses further revealed variability in performance: among female participants, the two systems agreed in 7 of 10 cases (70%) (six robust and one frail), whereas agreement was higher among males, with 10 of 11 cases (91%) classified identically (nine robust and one frail) (Cohen’s kappa (κ), (Female) κ = 0.211, *n* = 10, *p* = 0.490; Cohen’s kappa (κ), (Male) κ = 0.621, *n* = 11, *p* = 0.026).

Table 4 presents the criterion validity analysis comparing the high-accuracy home-monitoring system with three established frailty measures: the Fried’s Frailty Phenotype criteria, the CFS, and the EFS, using Spearman’s rho correlations for 21 participants. The IoT Frailty Platform showed a very strong and statistically significant association with the Fried’s Frailty Phenotype criteria, and a strong, statistically significant association with the CFS. Its correlation with the EFS was moderate to strong and statistically significant.

## 4. Discussion

This study aimed to design and validate a high-accuracy home-based monitoring system for assessing and tracking frailty in older adults. The system incorporated off-the-shelf solutions based on zero-effort technologies to facilitate usability for older adults, including individuals with mild cognitive impairment. To capture a representative sample of the intended end-user population, 21 adults aged 21 to 90 years (mean age = 51.86, SD = 26.48 years) participated in the study, encompassing both robust individuals and those with varying degrees of frailty. Frailty status was determined using the Fried’s Frailty Phenotype criteria as the gold standard, which includes five components: unintentional weight loss, exhaustion, low physical activity, weakness, and slowness [14]. We found that the proposed home-monitoring system demonstrated very strong agreement with the Fried’s Frailty Phenotype criteria, strong agreement with the CFS, and moderate to strong agreement with the EFS, all statistically significant. Overall, these results confirm the system’s strong concurrent validity for assessing and monitoring frailty in older adults.

The Frailty Phenotype criteria proposed by Fried and colleagues have been the most commonly used ground truth for assessing frailty in previous studies [11,55]. We also adopted this phenotype approach, as it aligns well with the validation of IoT-based monitoring systems. Alternative measures based on the deficit accumulation model assess frailty through the presence of diagnosed medical conditions (e.g., cancer, osteoporosis, hypertension, congestive heart failure), which typically require physician evaluation and are less feasible for automated home-based assessments. In contrast, the five-criterion Fried’s Frailty Phenotype can be operationalized through home-based sensors to monitor physical, cognitive, or behavioral changes indicative of frailty progression.

When compared with this benchmark, the frailty classification generated by our home-monitoring system demonstrated moderate to substantial and statistically significant agreement, as well as strong and statistically significant correlations with the Fried’s Frailty Phenotype criteria. These results support the concurrent validity of the home-monitoring system proposed. However, several discrepancies between the two systems warrant attention. Although both approaches identified the same number of individuals classified as robust, the high-accuracy home-based monitoring system identified fewer participants as pre-frail (nine cases compared to eleven identified by the standard measures) and more participants as frail (five cases compared to three identified using Fried’s Frailty Phenotype). Despite these discrepancies, the system’s ability to correctly classify most individuals as either robust or pre-frail is encouraging. Early and accurate identification of pre-frailty is clinically important, as it provides a critical window for preventive interventions that may slow down or mitigate progression to frailty. These findings therefore suggest meaningful clinical potential for the high-accuracy home-based monitoring system in supporting early detection and proactive management of frailty.

The frailty criteria that contributed most to discrepancies between the standard assessments and the home-monitoring system were slowness (three additional cases), weakness (two additional cases), and low physical activity (two additional cases). These differences may reflect measurement error or variation introduced by instrumentation and protocol implementation. For instance, slight timing inconsistencies among research assistants could have affected the standard measures for slowness. Likewise, despite both dynamometers being calibrated, the K-Grip^®^ device, the IoT dynamometer used to assess weakness (grip strength), produced lower readings in 89% of participants (17/19) and higher readings in 11% (2/19), with an average difference of 4.6 kg. These discrepancies may be attributable to differences in physical design between the K-Grip^®^ and the standard dynamometer, which could have influenced grip force generation for some participants.

Regarding low physical activity, while Fried’s Frailty Phenotype criteria define this criterion using weekly caloric expenditure derived from self-reported questionnaires, our sensor-based classification was based on the total duration (in seconds) required to complete two instrumental activities of daily living tasks. Participants whose performance time exceeded the 80th percentile were classified as frail, representing the slowest 20% of the sample, analogous to the lowest 20% of weekly caloric expenditure in frailty cut-off values from questionnaire-based measures such as the CHAMPS. Although the 80th percentile of task-completion time and the lowest quintile of weekly caloric expenditure are not physiologically equivalent, they are conceptually comparable in that both identify approximately the lowest-performing 20% of individuals on physical activity. Further, the two additional frail cases identified by the home-monitoring system may be explained by the system’s use of time-based metrics, which are likely more sensitive to early or subtle declines in activity performance. Similar findings have been reported by Park and colleagues, who observed that sensor-derived parameters such as percentage of time standing, walking cadence, and longest walking bout were associated with frailty phenotypes and could detect pre-frailty before full manifestation [56]. Additionally, our home-based monitoring reflects real-world and real-time performance rather than self-reported activity, providing greater ecological validity. This approach can reveal functional limitations, such as slower task performance during moderate-intensity physical activities, that questionnaires may not capture. Recent research further supports the potential of digital biomarkers and information and communication technologies for the early detection of frailty in home settings, consistent with our finding that the developed home-monitoring system identified a greater number of pre-frail and frail participants than traditional assessments [55].

Our study also introduces a paradigm shift from conventional sensor-based frailty monitoring approaches that focus primarily on mobility-related indicators—such as gait speed, postural transitions, and step counts—toward activity-based monitoring that captures how older adults perform meaningful daily tasks. Whereas traditional sensor systems typically quantify movement or mobility parameters in isolation, our approach emphasizes functional performance within real-life contexts. This shift acknowledges that frailty extends beyond physical mobility to encompass the interaction of physical and cognitive factors during everyday activities. By examining how individuals perform routine tasks rather than how they move in controlled settings, our home-monitoring system provides a more ecologically valid and functionally meaningful view of frailty, one that better reflects the capacities required for independent living and everyday participation.

The findings of this study provide insight into the usability of the home-based monitoring system across participants with diverse cognitive, educational, and demographic characteristics, while also revealing sex-related patterns consistent with previous frailty research. All participants were able to effectively use the developed home-based monitoring system, even though nine (43%) had an educational level below a university degree, and nearly half (52.4%) were older than 60 years of age. Among these older participants, five (45%) had SLUMS scores between 21 and 26, suggesting possible mild cognitive impairment. These findings highlight the system’s usability across individuals with diverse cognitive and educational backgrounds, supporting its feasibility for broad use among older adults, including those with mild neurocognitive disorders.

Although the overall sample was relatively balanced by sex (11 males and 10 females), most inpatient participants were female (7 of 9, 78%), consistent with patterns observed in previous information and communication technologies-based frailty studies, where women tend to be overrepresented [11], which may stem from the sex differences in the prevalence frailty itself. Furthermore, in our sample, 7 of the 10 older adults (70%) classified as pre-frail or frail according to Fried’s Frailty Phenotype criteria were female. This finding aligns with the well-documented “male–female health–survival paradox,” whereby women live longer than men but experience higher levels of frailty, disability, and poorer health despite lower mortality at all ages [57]. Consequently, females constitute a particularly relevant and accessible population for frailty research, as they tend to survive longer but are more likely to exhibit characteristics associated with frailty.

To contextualize the selection of the indoor positioning component of our home-monitoring system, it is important to compare the range of available technologies, their respective trade-offs, and the rationale behind choosing UWB as the most suitable solution for both clinical and home applications. The most common technologies currently used for indoor positioning systems include Wi-Fi-based positioning systems, Bluetooth Low Energy solutions, Radio Frequency Identification systems, and UWB technology. Each of these approaches presents specific trade-offs between accuracy, coverage range, and cost. Radio Frequency Identification systems achieve the highest accuracy, with errors below 0.1 m; however, their very short range (typically under 1 m) requires a dense network of readers, making large-scale installations complex and expensive. UWB technology also offers high accuracy (errors below 0.3 m) and the longest range (up to 150 m), though at a higher cost and power consumption—tracked devices cost approximately €100–150, and fixed receivers around €120 each. Bluetooth Low Energy systems, in contrast, are valued for their low cost and energy efficiency, with acceptable accuracy (~1 m error), although their limited range (20–30 m) and the need for additional beacon deployment may constrain large installations. Finally, WiFi-based systems are widely available and cost-effective due to existing infrastructure, but their accuracy is limited (typically 5–15 m) unless supplemented with numerous access points, which increases costs [58].

Building on this technological landscape, our research team previously evaluated the accuracy of a UWB tracker for in-home positioning among older adults [59]. In that study, the team tested multiple configuration parameters, including sampling rate, anchor placement, and line-of-sight conditions, compared to manufacturer-recommended settings. The results demonstrated that localization accuracies remained within 14 cm across configurations. Static and dynamic accuracy tests were then performed in both a motion-capture laboratory and the Independent Living Suite. Mean localization accuracies were 7.0 ± 11.1 cm in the laboratory and 27.3 ± 12.9 cm in the ILS. During dynamic testing, accuracies averaged 19.1 ± 1.6 cm for rolling motions and 20.5 ± 4.2 cm for walking, confirming that the system met the 30 cm accuracy threshold typically targeted for indoor localization.

Given these trade-offs and prior validation results, UWB technology was selected for the present study as a component of the high-accuracy home-monitoring system because it offers a balance of accuracy and coverage suitable for both clinical and home applications. First, UWB’s high accuracy makes it well-suited for hospital-to-home transitions, allowing healthcare professionals to make informed discharge decisions based on frailty status and mobility performance. Second, while UWB is not the least expensive option, its superior accuracy enhances the clinical relevance and reliability of frailty assessment. Its high accuracy may facilitate advocacy by healthcare professionals, such as occupational therapists, for funding programs that enable the installation of such systems in older adults’ homes, contrasting with the limited utility of low-accuracy monitoring systems in clinical decision-making. Moreover, continuous UWB-based monitoring of declines in physical, cognitive, or emotional functioning could play a critical role in preventing undesirable health outcomes, such as bone fractures due to falls, dehydration, or death resulting from an inability to move between rooms due to severe weakness.

The results for the exhaustion criterion indicate that the interaction method implemented with the Bluetooth Speakerphone AIRHUG 01^®^ was not valid for capturing self-reported exhaustion as defined by Fried’s Frailty Phenotype. Although this approach was validated in a previous study involving younger participants, our findings showed no correlation or agreement with the two CES–D items. Several factors may explain this discrepancy, including differences in the hardware and degree of control over the voice-interaction system. Bian and colleagues used a Raspberry Pi–based smart speaker that allowed full programming control over the dialogue and system responses [9]. In our case, our original plan to use commercially available personal voice assistants (e.g., Amazon Alexa, Google Assistant, Apple Siri) was not permitted due to hospital restrictions. As a result, we employed the AIRHUG 01^®^ Bluetooth speakerphone, with data collected and stored locally. This system frequently misinterpreted participants’ responses, leading to inaccuracies that undermined its validity relative to the ground-truth CES–D items. In addition, a few participants reported that the smart speaker’s responses were noticeably slow, creating unexpected delays between questions and answers.

This limitation appears to arise from the specific implementation of the voice-interaction system rather than from the broader concept of voice-based assessment. Future studies should explore alternative platforms that provide greater control, higher speech-recognition accuracy, faster processing, and more naturalistic conversational capabilities, as the scripted dialogue used in this study—although validated previously—was not well received by older adults. Given that older adults often report personal voice assistants as easy to use and well integrated into their daily routines, research conducted in participants’ homes—where such commercially available systems can be used without the restrictions of a healthcare facility—may be particularly valuable. In home environments, these systems may offer more promising opportunities for capturing indicators of exhaustion through naturalistic voice interactions [60].

Finally, our high-accuracy home-monitoring system demonstrated moderate to substantial agreement and moderate to strong correlations with Fried’s Frailty Phenotype criteria, both statistically significant. These results are difficult to directly contrast with previous research. Earlier studies reporting higher correlations or agreement values typically examined individual sensor outputs rather than overall frailty detection. For example, a recent study compared the number of door entry or exit events captured by video versus motion sensors, or weight values obtained from traditional and IoT-enabled scales [9]. In contrast, our approach assessed the concurrent validity of frailty classification itself by comparing the frailty score assigned to each criterion between the gold standard reference measure (i.e., the Fried’s Frailty Phenotype criteria) and the high-accuracy home-monitoring system. Another factor limiting comparability is the growing use of machine learning approaches in recent studies, which employ automated feature extraction and classification algorithms that differ substantially from our analytic methods [55,61]. Nonetheless, our findings are promising and support the feasibility of home-based monitoring systems as valid tools for frailty assessment and monitoring.

### Limitations

This study has several limitations that should be acknowledged. First, the relatively small and heterogeneous sample (*n* = 21) limits the generalizability of the findings, as variability in age and health status may have influenced the observed relationships. Second, the monitoring period was short, providing only a snapshot of performance rather than capturing potential trajectories in frailty status over time. Third, minor measurement inconsistencies may have occurred due to differences between devices and procedures; for instance, two cases of missing data were recorded for grip strength with the standard method, and small design differences in the dynamometer handles may have affected comparability. Future studies should investigate strategies to reduce these discrepancies. For an improved version of the system, this may include adding calibration steps, testing different equipment brands, since values may vary by brand, model, and design, and applying device-specific adjustments such as correction factors or tailored cut-off points for each IoT device. We did not implement these adjustments in the current study, as the inconsistencies between clinical and IoT devices represent an important finding that can inform future development. Moreover, the authors expected some level of discrepancy given that clinical instruments are calibrated for biomedical use, whereas most IoT devices may not be classified or regulated as medical equipment. A fourth limitation of this study was the performance of the Bluetooth Speakerphone AIRHUG 01^®^ used to capture self-reported exhaustion from Fried’s Frailty Phenotype criteria. The device showed suboptimal accuracy in recognizing verbal responses. Although commercially available smart speakers such as Amazon Alexa, Google Assistant, or Apple Siri may offer more robust voice recognition capabilities, their use was restricted due to institutional policies and patient data privacy regulations within the hospital setting. Fifth, participants were volunteers, which may have introduced selection bias toward individuals more comfortable with technology or in better overall health. Sixth, including healthy younger adults may affect threshold selection for frailty detection and limit generalizability to the intended target population of older adults at risk of frailty. However, based on Fried’s Frailty Phenotype, one older adult (0.9% of older participants) was classified as robust, while four younger adults (40% of younger participants) were classified as pre-frail or frail. This pattern is consistent with evidence showing that, although frailty is more prevalent in older adults, younger individuals can also present pre-frail or frail status [62]. Identifying and monitoring frailty indicators earlier in adulthood is increasingly recognized as valuable, as it allows for tailored behavioral interventions that support healthier lifestyles and may slow frailty progression over time. Future research with larger samples will be important to refine threshold values for the IoT-derived indicators of slowness and low physical activity, whose cut-offs in this study were calculated from sample-specific distributions. Seventh, since we used CHAMPS instead of the MLTPAQ, some variation in physical activity and kcal/week estimates may reflect differences between instruments. Finally, for inpatient participants, the CHAMPS questionnaire relied on self-reported physical activity prior to hospitalization, which may have been affected by memory recall errors and reduced cognitive accuracy.

Next steps should focus on further testing and refining this high-accuracy home-monitoring system to enhance its accuracy, applicability, and clinical utility. Future work that includes longitudinal studies with extended home-based monitoring periods would evaluate the system’s capacity to detect changes in frailty status and predict adverse health outcomes over time. Implementing these studies in real residential environments, rather than simulated home-like hospital settings, will allow examination of the system’s performance under everyday familiar or personalized living conditions and environmental variability. Additional refinement of sensor calibration, data-collection protocols, and integration with other health data could further reduce measurement error and improve standardization across devices. Future work that also explores the development of continuous and weighted frailty scoring methods within the high-accuracy home-monitoring system would enhance sensitivity to subtle, progressive changes in functional performance and to better reflect the multidimensional nature of frailty. Furthermore, by incorporating machine learning approaches, we could strengthen classification accuracy, personalize feedback, and improve scalability for clinical and home applications. Finally, an examination of the usability and adoption among older adults with advanced cognitive or physical impairments and limited digital literacy, could ensure the system remains inclusive and suitable for real-world deployment.

## 5. Conclusions

This study demonstrated the feasibility and concurrent validity of a high-accuracy home-monitoring system for assessing and tracking frailty in older adults. By integrating off-the-shelf, zero-effort technologies, the system successfully operationalized the five criteria of Fried’s Frailty Phenotype in a realistic, home-like environment. The system showed a very strong and statistically significant correlation with the Fried’s Frailty Phenotype criteria, a strong correlation with the CFS, and a moderate to strong correlation with the EFS, supporting its strong concurrent validity for frailty assessment with other well-known frailty scales used in clinical practice.

Beyond validation, this study advances the field by shifting from mobility-centric frailty assessment and monitoring toward activity-based assessment that reflects how older adults perform meaningful daily tasks. This approach enhances ecological validity and provides a richer understanding of frailty as a multidimensional construct encompassing physical and cognitive domains. The findings also demonstrate the system’s usability among participants with diverse cognitive and educational profiles, suggesting its potential applicability for widespread use, including among individuals with mild cognitive impairment.

The results underscore the promise of high-accuracy, sensor-based home monitoring for enabling continuous, objective, and unobtrusive frailty assessment and monitoring. Such systems can support early identification of frailty progression, timely clinical interventions, and data-driven decision-making to promote independent living and reduce healthcare burden. Together, these developments could lay the foundation for next-generation smart home systems that transform frailty management and support healthy aging in place.

## Figures and Tables

**Table 1:** Sex- and BMI-specific grip strength thresholds used to classify frailty for weakness criterion.

Sex	BMI ≤ 24	BMI 24.1–26	BMI 26.1–28	BMI > 28
Male	≤29 kg	≤30 kg	≤31 kg	≤32 kg
Female	≤17 kg	≤17.3 kg	≤18 kg	≤21 kg

**Table 2:** Participants’ demographic and clinical data [*n* = 21].

Variables	Biological Sex		
	Overall (*n* = 21)	Male (*n* = 11)	Female (*n* = 10)
	Mean (SD)		
Age (Min = 21, Max = 90)	51.86 (26.48)	43.45 (25.41)	61.10 (25.68)
Sex	*n* (%)		
Male	11 (52.4)	11 (52.4)	0
Female	10 (47.6)	0	10 (47.6)
Body Composition	Mean (SD)		
Weight (Kgs) (Min = 52.90, Max = 131.00)	78.26 (19.69)	79.21 (16.40)	77.21 (23.67)
Height (cm) (Min = 153.00, Max = 194.50)	170.74 (10.72)	176.18 (9.59)	164.76 (8.78)
BMI (Min = 18.49, Max = 48.28)	27.21 (7.10)	26.24 (5.42)	28.26 (8.78)
Age group	*n* (%)		
≤60	10 (47.6)	7 (63.6)	3 (30.0)
61–74	6 (28.6)	3 (27.3)	3 (30.0)
75–84	3 (14.3)	0	3 (30.0)
85–94	2 (9.5)	1 (9.1)	1 (10.0)
Education	*n* (%)		
High school	6 (28.6)	3 (27.3)	3 (30.0)
Trade school	3 (14.3)	1 (9.1)	2 (20.0)
Bachelor’s	7 (33.3)	4 (36.4)	3 (30.0)
Master’s	4 (19.0)	2 (18.2)	2 (20.0)
Doctorate or above	1 (4.8)	1 (9.1)	0
English as First Language	*n* (%)		
Yes	19 (90.5)	9 (81.8)	10 (100)
No	2 (9.5)	2 (18.2)	0
Residential Status	*n* (%)		
Community alone	5 (23.8)	2 (18.2)	3 (30.0)
Community with others	14 (66.7)	9 (81.8)	5 (50.0)
Retirement home or assisting living	2 (9.5)	0	2 (20.0)
Handedness	*n* (%)		
Left	1 (4.8)	1 (9.1)	0
Right	20 (95.2)	10 (90.9)	10 (100)
Polypharmacy	*n* (%)		
Yes	10 (52.4)	3 (27.3)	7 (70.0)
No	11 (47.6)	8 (72.7)	3 (30.0)
Inpatient Status	*n* (%)		
Yes	9 (42.9)	2 (18.2)	7 (70.0)
No	12 (57.1)	9 (81.8)	3 (30.0)
Baseline Assessments	Mean (SD)		
SLUMS (Min = 21, Max = 30)	27.81 (3.01)	28.82 (2.23)	26.70 (3.46)
Barthel Index (Min = 15, Max = 20)	18.95 (1.59)	19.45 (1.51)	18.40 (1.58)
CES-D Scale total score (Min = 2, Max = 16)	6.29 (3.52)	6.64 (4.20)	5.90 (2.77)
Maximum Grip Strength (Kg)	Mean (SD)		
Dominant hand (Min = 20, Max = 49.09)	34.37 (9.90)	38.87 (8.91)	27.04 (6.73)
Non-dominant hand (Min = 10.00 Max = 51.81)	31.04 (11.98)	37.60 (9.46)	22.02 (8.97)

**Table 3:** Reliability and Validity: High-accuracy home-monitoring system compared to the Fried’s Frailty Phenotype criteria (*n* = 21).

	Descriptive Statistics		Reliability		Criterion Validity	
Fried Frailty Phenotype Criterion	Ground Truth	High-Accuracy home-Monitoring System	Cohen’s Kappa	*p*	Spearman ρ (df)	*p*
Unintentional weight lost (Shrinking)	Self-Report Yes [*n* = 6, 28.6%] No [*n* = 15, 71.4%]	Self-Report using smart speaker (Bluetooth Speakerphone AIRHUG 01^®^) Yes [*n* = 6, 28.6%] No [*n* = 15, 71.4%]	1.000	Not applicable	1.000 (19)	Not applicable
Low physical activity	CHAMPS Not Limited, or Little Limited [*n* = 18, 85.7%] Limited A Lot [*n* = 3, 14.3%]	UWB Monitoring (Pozyx^®^) of PASS Items (Time) Not Limited, or Little Limited [*n* = 16, 76.2%] Limited A Lot [*n* = 5, 23.8%]	0.391	0.06	0.411 (19)	0.064
Low endurance (Exhaustion)	CES-D Questionnaire 0–2 Days [*n* = 16, 76.2%] 3–7 Days [*n* = 5, 23.8%]	Smart speaker (Bluetooth Speakerphone AIRHUG 01^®^) CES-D 0–2 Days [*n* = 18, 85.7%] 3–7 days [*n* = 3, 14.3%]	0.087	0.676	0.091 (19)	0.694
Weakness (Grip strength)	Manual Dynamometer <20% Weaker [*n* = 15, 71.4%] >20% Weaker [*n* = 4, 19.0%] Missing data [*n* = 2, 1%]	K-Grip^®^ device Dynamometer <20% Weaker [*n* = 14, 66.7%] >20% Weaker [*n* = 7, 33.3%]	0.855	<0.001	0.864 (19)	<0.001
Slowness (Walking time)	15 feet walk time—Timer Normal [*n* = 15, 71.4%] Slower [*n* = 6, 28.6%]	15 feet walk time—UWB (Pozyx^®^) Normal [*n* = 9, 42.9%] Slower [*n* = 12, 57.1%]	0.462	0.012	0.548 (19)	0.010
Reliability (Parallel-Forms): Fried’s Frailty Phenotype criteria—High-accuracy home-monitoring system Classification (Dichotomized)						
Frailty Classification (Dichotomized)	Not Frail (Robust, Pre-Frail) Fried Phenotype: *n* = 18, 85.7% Frail (Frail, Very frail) Fried Phenotype: *n* = 3, 14.3%	Not Frail (Robust, Pre-Frail) High-accuracy home-monitoring system: *n* = 16, 76.2% Frail (Frail, Very frail) High-accuracy home-monitoring system: *n* = 5, 23.8%	0.696	<0.001	0.730 (19)	<0.001
Reliability (Parallel-Forms): Fried’s Frailty Phenotype criteria—High-accuracy home-monitoring system Classification (Original total score)						
Frailty Classification (Original total score)	Robust (Frailty score = 0) *n* = 7, 33.3% Pre-Frail (Frailty score = 1–2) *n* = 11, 52.4% Frail (Frailty score = 3–4) *n* = 3, 14.3%	Robust (Frailty score = 0) *n* = 7, 33.3% Pre-Frail(Frailty score = 1–2) *n* = 9, 42.9% Frail (Frailty score = 3–4) *n* = 5, 23.8%	0.547	<0.001	0.715 (19)	<0.010

**Table 4:** Frailty scales: High-accuracy home-monitoring system—Frailty Scales (Criterion validity; *n* = 21).

Frailty Assessment	Statistic	1	2	3	4
1. High-accuracy home-monitoring system	Spearman ρ	--			
	*p*-value	.			
2. Fried’s Frailty Phenotype	Spearman ρ	0.843	--		
	*p*-value	<0.001	.		
3. Clinical Frailty Scale	Spearman ρ	0.662	0.620	--	
	*p*-value	<0.001	0.003	.	
4. Edmonton Frailty Scale	Spearman ρ	0.599	0.531	0.826	--
	*p*-value	0.004	0.013	<0.001	.

## Data Availability

The data sets generated and analyzed during this study are not publicly available due to the limitations imposed by the study’s ethical approval, but are available from the corresponding author on reasonable request.

## Supplementary Materials

The following supporting information can be downloaded at: https://www.mdpi.com/article/10.3390/s26010113/s1, Table S1: Participants’ technology literacy; Table S2: Participants’ frailty profile.

## Abbreviations

The following abbreviations are used in this manuscript:

UWB	Ultra-Wideband
IoT	Internet of Things
PEAR	Product Evaluation and Application Research
PASS	Performance Assessment of Self-Care Skills
CES-D	Center for Epidemiological Studies–Depression
CHAMPS	Community Healthy Activities Model Program for Seniors
MET	Metabolic Equivalent of Task
BMI	Body Mass Index
SLUMS	Saint Louis University Mental Status
CFS	Clinical Frailty Scale
EFS	Edmonton Frailty Scale
SPSS	Statistical Package for the Social Sciences
ICC	Intraclass Correlation Coefficient
SD	Standard Deviation
df	Degrees of Freedom
