# Supplementary material for: High-Accuracy Indoor Positioning and Smart Home Technologies for Assessing and Monitoring Frailty in Older Adults
Source: Sensors (Basel). 2025 Dec 24;26(1):113. doi: 10.3390/s26010113 (PMC12787328; doi:10.3390/s26010113)
Supplement: Supplementary file 1 [file sensors-26-00113-s001.zip › sensors-4001921-supplementary.pdf]

Supplementary Materials

Participants' technology literacy and frailty profile.

Table S1. Participants' technology literacy [n=21]

**Variables**	
Smartphones	***n (%)***
**Daily**	18 (85.7)
**Twice weekly**	0
**Weekly**	0
**1-2 times per month**	0
**Less than once per month**	0
**Few times per year**	0
**Never**	1 (4.8)
**Missing value**	2 (9.5)
Smart TV	***n (%)***
**Daily**	10 (47.6)
**Twice weekly**	0
**Weekly**	0
**1-2 times per month**	1 (4.8)
**Less than once per month**	0
**Few times per year**	3 (14.3)
**Never**	5 (23.8)
**Missing value**	2 (9.5)
Smart watch	***n (%)***
**Daily**	4 (14.3)
**Twice weekly**	0
**Weekly**	0
**1-2 times per month**	0
**Less than once per month**	*0*
**Few times per year**	2 (9.5)
**Never**	12 (57.1)
**Missing value**	3 (14.3)
Tablets	***n (%)***
**Daily**	6 (28.6)
**Twice weekly**	2 (9.5)
**Weekly**	0
**1-2 times per month**	0
**Less than once per month**	2 (9.5)
**Few times per year**	2 (9.5)
**Never**	7 (33.3)
**Missing value**	2 (9.5)
Computer	***n (%)***
**Daily**	17 (81)
**Twice weekly**	2 (9.5)
**Weekly**	1 (4.8)
**1-2 times per month**	0

**Less than once per month**	0
**Few times per year**	0
**Never**	1 (4.8)
**Missing value**	0
Smart home devices	***n (%)***
**Daily**	6 (28.6)
**Twice weekly**	*0*
**Weekly**	1 (4.8)
**1-2 times per month**	0
**Less than once per month**	0
**Few times per year**	11 (52.4)
**Missing value**	2 (9.5)

Table S2. Participants' frailty profile. [n=21]

**Frailty scales**	
Fried Frailty Phenotype	***n (%)***
**Robust**	7 (33.3)
**Pre-frail**	11 (52.4)
**Frail**	3 (14.3)
**Very frail**	0
Edmonton Frailty Scale	***n (%)***
**Not frail**	17 (80.9)
**Vulnerable**	4 (19.1)
**Mild frailty**	0
**Moderate frailty**	0
**Severe frailty**	0
Clinical Frailty Scale	***n (%)***
**Very fit**	6 (26.6)
**Well**	6 (28.6)
**Managing well**	4 (19.0)
**Vulnerable**	4 (19.0)
**Mildly frail**	1 (4.8)
**Moderately frail**	0
**Severely frail**	0
**Very severely frail**	0
**Terminally ill**	0
